# Tiny Is Mighty: Seagrass Beds Have a Large Role in the Export of Organic Material in the Tropical Coastal Zone

**DOI:** 10.1371/journal.pone.0111847

**Published:** 2014-11-11

**Authors:** Lucy G. Gillis, Alan D. Ziegler, Dick van Oevelen, Cecile Cathalot, Peter M. J. Herman, Jan W. Wolters, Tjeerd J. Bouma

**Affiliations:** 1 Spatial Ecology Department, Royal Netherlands Institute for Sea Research (NIOZ), Yerseke, Zealand, The Netherlands; 2 Geography Department, National University of Singapore (NUS), Singapore, Singapore; 3 Ecosystems Studies Department, Royal Netherlands Institute for Sea Research (NIOZ), Yerseke, Zealand, The Netherlands; 4 Laboratoire Environnement Profond (LEP), French Research Institute for Exploitation of the Sea, Polouzane, Brittany, France; 5 Department of Biology, University of Antwerp, Antwerp, Flanders, Belgium; Scottish Association for Marine Science, United Kingdom

## Abstract

Ecosystems in the tropical coastal zone exchange particulate organic matter (POM) with adjacent systems, but differences in this function among ecosystems remain poorly quantified. Seagrass beds are often a relatively small section of this coastal zone, but have a potentially much larger ecological influence than suggested by their surface area. Using stable isotopes as tracers of oceanic, terrestrial, mangrove and seagrass sources, we investigated the origin of particulate organic matter in nine mangrove bays around the island of Phuket (Thailand). We used a linear mixing model based on bulk organic carbon, total nitrogen and δ^13^C and δ^15^N and found that oceanic sources dominated suspended particulate organic matter samples along the mangrove-seagrass-ocean gradient. Sediment trap samples showed contributions from four sources oceanic, mangrove forest/terrestrial and seagrass beds where oceanic had the strongest contribution and seagrass beds the smallest. Based on ecosystem area, however, the contribution of suspended particulate organic matter derived from seagrass beds was disproportionally high, relative to the entire area occupied by mangrove forests, the catchment area (terrestrial) and seagrass beds. The contribution from mangrove forests was approximately equal to their surface area, whereas terrestrial contributions to suspended organic matter under contributed compared to their relative catchment area. Interestingly, mangrove forest contribution at 0 m on the transects showed a positive relationship with the exposed frontal width of the mangrove, indicating that mangrove forest exposure to hydrodynamic energy may be a controlling factor in mangrove outwelling. However we found no relationship between seagrass bed contribution and any physical factors, which we measured. Our results indicate that although seagrass beds occupy a relatively small area of the coastal zone, their role in the export of organic matter is disproportional and should be considered in coastal management especially with respect to their importance as a nutrient source for other ecosystems and organisms.

## Introduction

The tropical coastal zone is composed of ecosystems emerging from the open ocean, extending over coral reefs and seagrass beds towards the tidal zone, which may include mangrove forests. This zone forms a well-structured and gradual interface between the land and the sea that contains some of the most productive and biogeochemically active ecosystems in the world [1]. Part of this productivity is maintained by particulate and dissolved organic matter (POM and DOM) inputs from both terrestrial and oceanic sources [2].

The importance of external inputs to the coastal zone and the exchanges between coastal ecosystems depends on various factors. For example, the quantity of terrestrial organic matter (OM) input to the coastal zone has been shown to depend on the size of the catchment area and the land-use within [3]. Tidally-dominated estuaries have large exchanges of water with the ocean, and therefore, receive ocean-derived DOM and POM [4]–[6]. The recognized mangrove outwelling hypothesis [7]–[8] postulates that export of mangrove-derive OM supports adjacent ecosystems and food webs.

Another potentially important OM source and sink within the tropical coastal zone are seagrass beds. Seagrass plants trap suspended POM originating from both external sources and from leaf shedding inside the seagrass bed [9]–[10]. Trapping results directly from the physical structure of the bed and from settling induced by changes in the hydrodynamic conditions - both are related in part to plant density and leaf characteristics [11]–[12]. Once POM is deposited within seagrass beds, the matter is ‘protected’ from re-suspension, and consequently, most POM degrades within the bed, releasing dissolved nutrients that may in turn be taken up by the seagrass [13]–[15] or released to the water column [9], [16]. Seagrass beds however also export particulate nutrients via leaf shedding enhanced during strong hydrodynamic events and via marine herbivore consumption [17].

Due to the different ecosystems in the seascape, the high production of ecosystems and the potential for import and export from a variety of sources, it is challenging to constrain the contribution of the different resources to the exchange of or OM in the tropical coastal zone. One classical approach is to use a chemical tracer mixing model based on a combination of carbon to nitrogen ratios (C:N) with carbon and nitrogen stable isotope ratios (δ^13^C and δ^15^ N, respectively) [18]–[19]. Each of these tracers has a specific signature for each source (oceanic, seagrass, mangrove and terrestrial), which can be used to identify the contribution of the different sources in a mixture [18]–[19].

Investigations of source contributions in Southern Thailand and found that seagrasses contributed between 36–42% of OM to sediment trap samples [4]. Other studies in Gazi Bay, Kenya discovered that mangrove and seagrass matter dominated suspended POM in the water column [17], [20]. These studies indicate that contributions from individual sources likely depend on the local conditions. In this paper, we build upon the foundations of previous work to investigate the origin of POM in trap and suspended sediment samples in the coastal zone in Phang-nga Bay in southern Thailand. We studied nine sites with contrasting physical attributes of bays (width and length), of ecosystems (aerial extent and width) and of catchment areas (aerial extent and land use). This allowed us to gain a greater understanding of export of POM from different ecosystems at the tropical seascape scale. Specifically, we compared the amount of OM originating from seagrass beds versus mangrove, oceanic, and terrestrial sources across a number of different sub-habitats in the region, comparing the contributions to their respective surface areas.

## Methods

### Ethics statement

The work was conducted in collaboration with Rajabhat University (Phuket) on public lands. No permit was required for sampling. No live samples were collected.

### Study Area and Physical attributes

**Figure 1:**
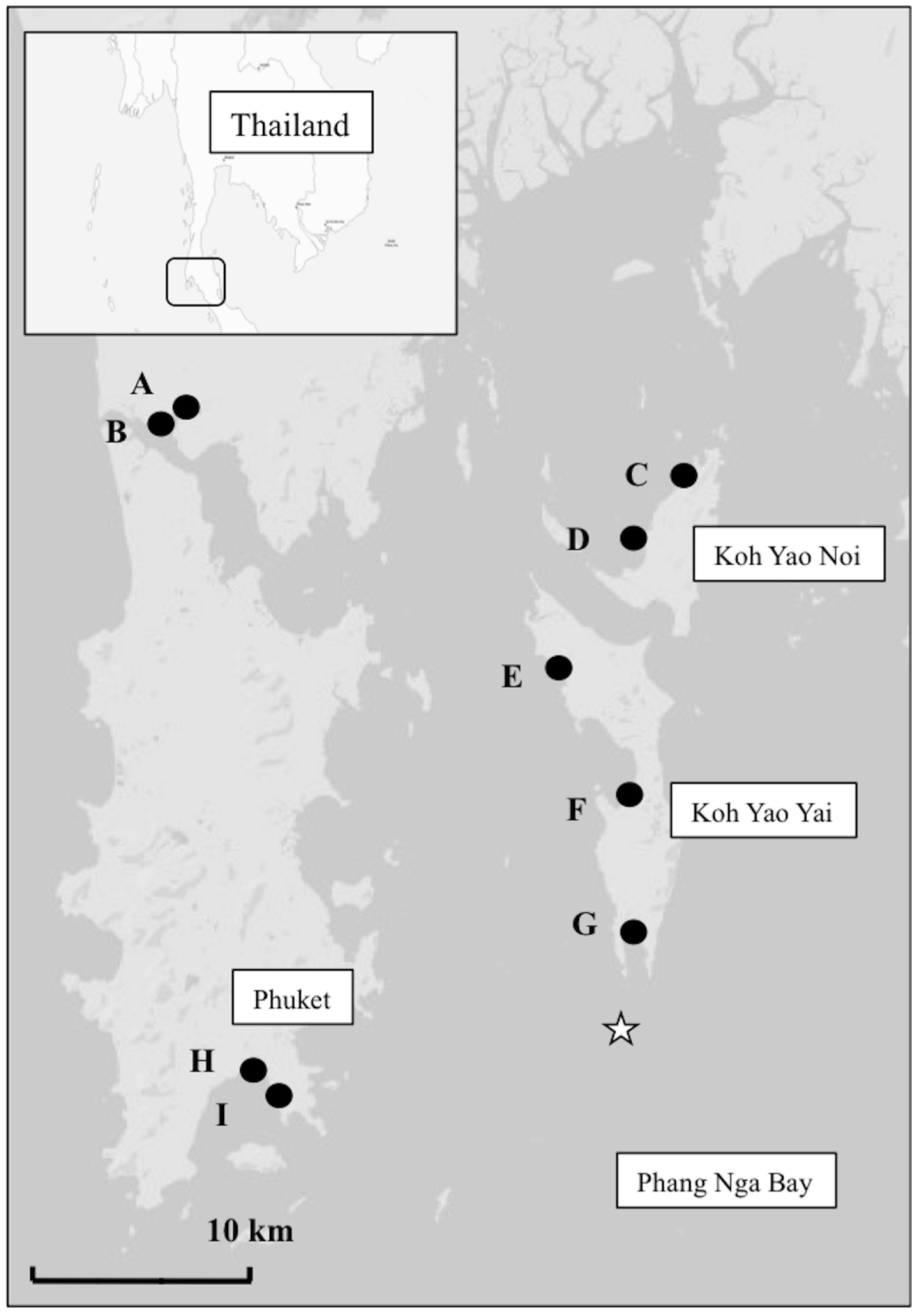
Map showing location of the nine sampling sites (black circles) in Phang Nga Bay, southern Thailand. The white star represents the point oceanic samples where collected.

Within the Andaman Sea, along the southwest coast of Thailand is the province of Phuket, which borders Phang-nga bay (Figure 1). Our sampling sites are located in Phang-nga Bay, which is 68 km long (head to mouth) and has a surface area of 3000 km^2^. Mean tidal range is 1.8 m (Figure 1). The southern part of the bay is open to the Andaman Sea and the northwest area is open to the sea via the Pak Pra Inlet, which separates Phuket Island from the mainland (Figure 1). Estuarine salinity conditions dominate in the north, whilst marine conditions dominate in the south [21]. Circulation in the bay changes depending on the dry (May to October) or wet season (November to April), which alters the wind direction from northeast in the dry season to southwest in the wet season. Mean annual rainfall is about 2300 mm and mean temperature is 28°C.

Land-use around Phang-nga Bay changed from natural forest to initially tin mining (1600–1800) [21]. Other land-uses have gained greater importance such as rubber and palm oil plantations as well as shrimp (located on land) and fish farms (situated in coastal zones) [21]. In recent decades, rapid urbanisation related to tourism has occurred in Phuket.

Samples were collected from all the fully accessible mangrove sites located in the west of Phang-nga Bay, on the islands of Phuket, Yao Yai and Yao Noi. A total of 9 sites were sampled (Figure 1) during March 2011. All sites were outside the reach of direct river discharge and experienced a strong tidal exchange (tidal range 0.5–2.5 m) with the ocean. Therefore, all sites are classified as tidal mangroves [22].

### Sample collection and data processing

At each site we established transects beginning at the edge of the mangrove, extending towards the ocean through an adjacent intertidal seagrass patch. Samples were taken, when logistically possible, at distances of 0 (seaward edge of mangrove forest), 50, 100, 200, 300, 400, 500, 1000, 1500 and 2000 m along the transect, perpendicular to the seaward edge of the mangrove forest towards the open sea. Two types of samples were collected: trap samples of suspended matter above the bed (0.05 m) and suspended particulate matter in the water column. At least half of the sampling points (for trap and water samples) were located in the seagrass beds; the other half were taken at points landward or seaward of the bed, depending on the physical constraints of the different sites. Sediment traps at each point were secured to steel rods and anchored to the substrate at a height of 0.05 m above the sediment. They were emptied once after approximately 24 hours. On the day of trap installation, 2-liter surface water samples were taken at each point above the traps at the water surface during high tide.

Four POM sources were considered in this study: terrestrial vegetation, mangrove leaves, seagrass leaves and oceanic plankton. For the terrestrial vegetation, three replicates of 3–4 leaves from rice paddies, rubber trees (*Hevea sp.*) and native vegetation (*Delonix sp.* and *Terminalia sp.*) were collected on the island of Koh Yai. These were considered to be representative for terrestrial vegetation of the province. This matter represents the source value for the terrestrial OM used in the mixing model. Mangrove and seagrass leaves were collected during the trap deployment. Plankton and suspended particulate matter (SPM) samples were collected at a point believed to have the majority of oceanic influence (7°52.573′N and 98°35.635′E) and taken as proxy for oceanic matter. Plankton samples were collected with weighted nets of mesh sizes 400 µm and SPM with a five 1-litre water sample. The mean of the C and N content and δ^13^C and δ^15^ N value of each POM source was taken as the respective end-member to determine the contribution of each source to the OM along the transect.

All solid samples (sediment and leaves) were placed in separate sample bags, stored in a cooling box and transported immediately to the field laboratory where they were dried at 60°C for 48 hours. Water samples for SPM and plankton were filtered through pre-combusted glass fiber filters (GF/F, 0.7 µm pore diameter). The GF/F filters were dried at 60°C for 48 hours. Both solid material (sediment and leaves) and GF/F filters were packed in airtight containers and transported to the laboratory of Royal Netherlands Institute for Sea Research (NIOZ) for elemental and isotope analysis.

### Elemental and isotope analysis

Sediment and leaf material samples were ground for homogenisation. The trap, plankton and SPM samples were acidified to remove carbonates [23]; the leaf samples were not acidified. All samples were analysed for total organic carbon (TOC), total nitrogen (TN), and the isotopes δ^13^C and δ^15^N by means of elemental analysis isotope ratio mass spectrometry (EA-IRMS) using a Thermo Finnigan Flash 1112.

Stable isotope ratios are expressed as δ values (‰) relative to conventional standards (VPDB limestone for C and atmospheric N_2_ for N):
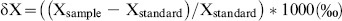
(1)where δX is either δ^13^C or δ^15^N; X_sample_ is the ^13^C/^12^C or ^15^N/^14^N ratio of the sample; X_standard_ is the ^13^C/^12^C or ^15^N/^14^N ratio of the standards (0.01118 for C and 0.00368 for N).

### Mixing model

We considered the following potential sources (Table 1): terrestrial vegetation (*Delonix sp.*, *Terminalia sp.* and *Hevea sp.*), mangrove leaves (*Rhizophora sp.*), seagrass leaves (*Enhalus sp.*, *Halodule sp.* and *Halophile sp.*) and oceanic production (plankton and SPM). Mean values were taken as end member for each source (Table 1). Only SPM and trap samples were analysed with the mixing model (as discussed below).

**Table 1:** Mean and standard error values of isotopes carbon isotope (δ^13^C), nitrogen isotope (δ^15^N), bulk organic carbon (C) and total nitogen (N) for terrestrial trees, mangroves trees, seagrass plants and oceanic sources.

Sources	Species	δ^13^C		δ^15^N		C		N		*n*
Group		Mean	SE	Mean	SE	Mean	SE	Mean	SE	
		(^o^/_oo_)		(^o^/_oo_)						
Terrestrial	*Havea sp.*	−31.8	0.5	0.4	0.001	46.8	0.2	4.2	0.04	3
	*Delomix sp.*	−30.1	0.5	0.6	0.001	46.9	0.3	2.6	0.2	3
	*Terminalia sp.*	−31.2	0.5	1.9	0.001	44.8	0.2	3.6	0.006	3
**Overall terrestrial**	**-**	**−31.0**	**0.4**	**1.0**	**0.0004**	**46.1**	**0.2**	**4.01**	**0.08**	**3**
**Overall Mangrove trees**	***Rhizophora sp.***	**−26.9**	**0.6**	**3.6**	**0.2**	**37.5**	**0.6**	**1.2**	**0.07**	**45**
Seagrass plants	*Halodule sp.*	−11.4	0.7	2.9	0.15	21.4	2.6	1.4	0.2	7
	*Halophile sp.*	−11.6	0.5	2.8	0.9	21.4	2.1	1.3	0.2	19
	*Enhalus sp.*	−10.1	0.3	5.2	0.3	25.6	1.6	1.9	0.1	17
**Overall seagrass plants**	**-**	**−11.0**	**0.6**	**3.6**	**0.2**	**21.7**	**1.4**	**1.5**	**0.1**	**3**
Oceanic	SPM	−24.1	0.13	18.3	3	1.2	0.07	0.2	0.01	3
	Plankton	−24	0.04	7.9	0.03	4.6	0.5	0.8	0.1	3
**Overall oceanic**	**-**	**−24.1**	**0.06**	**13.1**	**3.5**	**2.9**	**0.8**	**0.5**	**0.1**	**6**

The values for each source (highlighted) are the means of the different contributions (species for terresrtial, mangrove and seagrasses & SPM/plankton for oceanic). These values are defined as the end members (organic matter sources), which are used in the mixing model to determine the different mixture of fractions for organic matter. The organic matter was collected in the core, sediment trap and suspended sediment matter samples. *n,* is the number of samples and SE is standard error.

A linear mixing model was used to determine the contributions of the different sources to the SPM and trap samples [24]–[25]. The mixing model uses the means of δ^13^C, δ^15^N and the C:N ratios (corrected for differences in %C and %N between sources) of individual trap or SPM samples to determine the contribution (f) of each of the four sources (Table 1) to the mixture. The variation between replicates is small justifying the use of the means. The mixing model has the following four equations that are solved simultaneously to recover a unique solution of the four source contributions (f_oce_, f_ter_, f_man_, f_sea_): 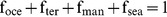
(2)

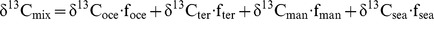
(3)

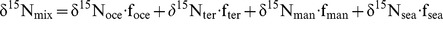
(4)

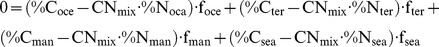
(5)


Where oce  =  oceanic, ter  =  terrestrial, man  =  mangrove trees and sea  =  seagrass plants.The model was implemented and solved in R [26], using the *lsei* function available in the LIM package [27]. Using the contribution for each source as returned by the mixing model, we calculated if each source was under-contributing or over-contributing compared to its relative surface:
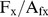
(6)


This index was not calculated for POM derived from oceanic sources, because no representative surface area for the ‘ocean’ could be defined. The contribution (F_x_; %) per source in each sample was then divided by the percent of total surface area (A_fx_) occupied by the particular system (catchment area-terrestrial, mangrove forest, seagrass bed). This gives a dimensionless number, for which 1 implies that the ecosystem contributes proportionally to its relative surface area; values below or above 1 indicates that the proportional contribution is lower or higher, respectively.

### Statistical analysis

From the results of equation 6 we used step-wise regression analysis to determine if the contribution of each resource was related to relevant physical aspects of the nine sites. For this analysis we only used the mixing model results at 0 and 100 m along the transect, as data for these distances were available across all nine sites. Contributions from terrestrial, mangrove and seagrass to SPM and trap samples were correlated with physical attributes of each site. The mangrove contribution was tested against area of mangrove forest (m^2^), area of bay (m^2^), urbanisation in the catchment area (%) and width of mangrove forest (m) (Table 2). Terrestrial contribution was analysed against catchment area (m^2^), area of bay (m^2^) and urbanisation in the catchment area (%) (Table 2). Finally, seagrass was tested with area of the seagrass bed (m^2^), area of bay (m^2^) and urbanisation in the catchment area (%) (Table 2).

**Table 2:** Physical description of the sites and the physical attributes of the marine ecosystems for each of the nine sites (A to I) and their associated bay.

Physical attributes	Site								
	A	B	C	D	E	F	G	H	I
Urbanized area (km^2^)	1.4	1.4	0.9	0.4	0.1	0.1	0.6	0.7	5.8
Mangrove forest area (km^2^)	3.3	3.3	10	0.6	7.5	3.2	2	9	4.7
Catchment area (km^2^)	29.6	29.6	3	0.2	0.6	0.7	1.5	2.1	0.4
Seagrass bed area (km^2^)	1	0.5	0.1	0.3	0.8	0.04	0.8	0.9	0.8
Width of bay (km)	0.97	0.97	NA	1.77	1.44	3.76	1.22	2	2
Length of bay (km)	1.47	1.47	NA	1.11	0.72	3.31	2.94	1.06	1.06
Width of mangrove forest (km)	3.4	3.4	0.06	0.71	1.18	0.89	1.84	0.96	0.31

NA means that these sites are situated in an open coast, all other sites are bays.

The physical characteristics (bay area, bay width/length), surface areas of the mangrove forests and seagrass beds were determined with ground truthing during the sampling campaign. Land use in the catchment area, width of the bay, and length of the bay were determined by analysing Quickbird, WorldView-1 and WorldView-2 satellite imagery. All statistical analysis was completed in R [26], where probabilities (p) <0.05 were considered significant.

## Results

**Figure 2:**
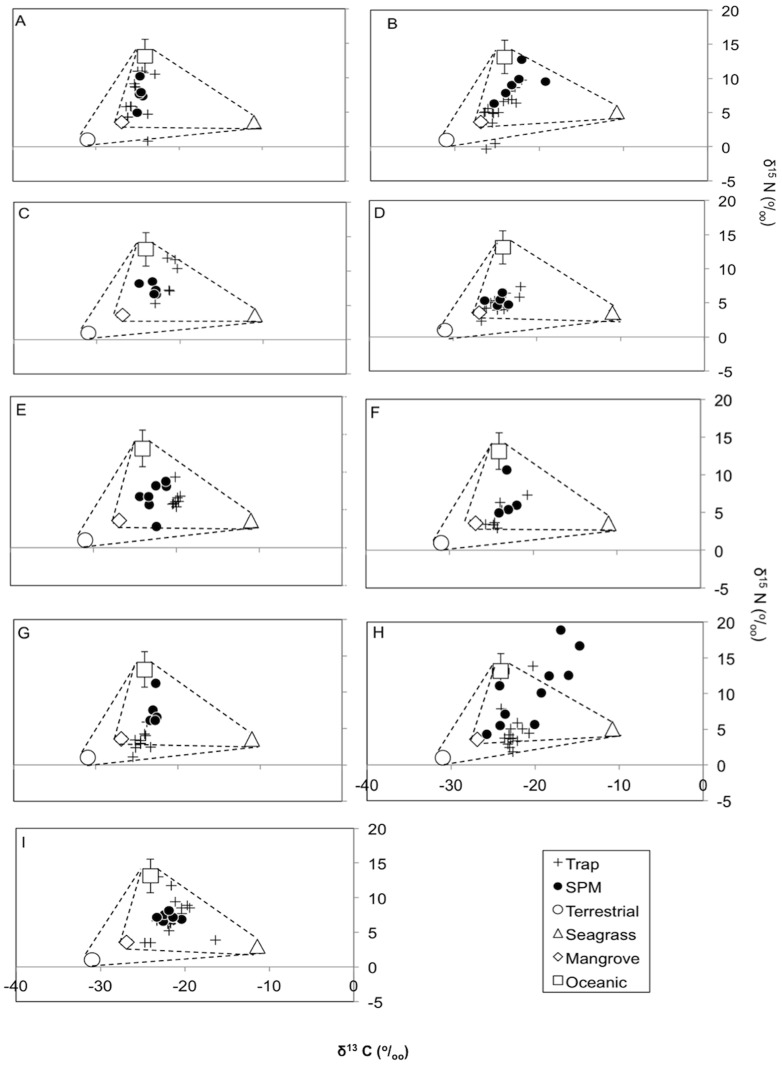
Carbon (δ^13^C ^o^/_oo_) and nitrogen (δ^15^N ^o^/_oo_) isotopic composition for organic matter from trap and suspended particulate matter (SPM) samples. The end members shown are: white circles represent the terrestrial value; white squares represent oceanic sources; white triangles represent seagrass plants; and white diamonds are mangrove trees. The trap and SPM samples are represented by crosses and black circles, respectively. Values for the end members are means (+/− SE). For the end members of the oceanic, terrestrial, seagrass plants and mangrove plants sources, n = 6, 9, 44 and 45, respectively. Note that this is a 2-dimensional representation of isotope values only. Table 3 shows results using all three components, i.e. carbon, nitrogen isotopes and C:N ratios.

All samples (both trap and SPM) along the gradient showed different contributions of the different sources of OM (Figure 2). At sites A and B, the signatures for the majority of samples were between those of terrestrial, mangrove and oceanic sources (Figure 2). The trap sample signatures from sites C and I were spread from seagrass to the oceanic end members (Figure 2). At sites D and F, the trap and SPM samples indicated a pattern from terrestrial and mangrove sources to the mid-point between all the sources (Figure 2). The majority of site E trap samples similarly plotted in the centre of the sources; and sites C, E and I SPM samples followed this pattern too (Figure 2). Terrestrial sources dominated trap samples from sites G and H, but in the case of SPM samples they where spaced between terrestrial and mangrove end numbers and the oceanic end number (Figure 2).

**Figure 3:**
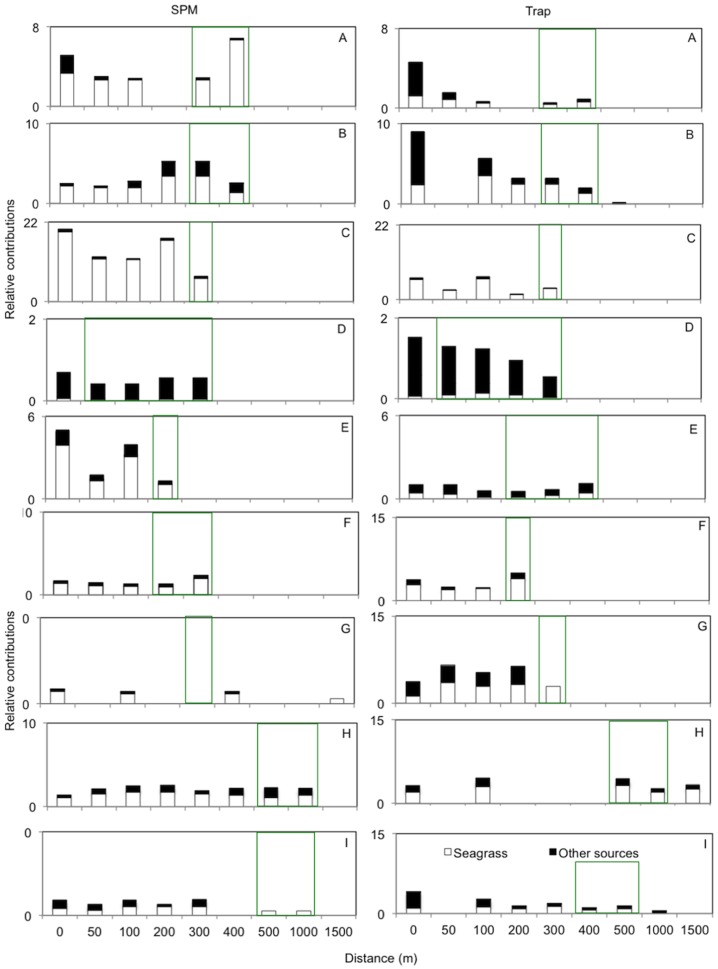
A dimensionless index indicating the relative contribution of seagrass plants and other sources (mangrove trees + terrestrial) for suspended particulate matter (SPM) and sediment trap samples (trap). The values were determined by dividing the mixing model contribution result (Table3) with the per cent of total surface area occupied by a particular ecosystem at each site. Distance (m) represents the distance along the length of the transect from the land (0 m) to the ocean for each site. SPM and trap are sample type, SPM is suspended particulate matter taken from the water column. Whilst trap represents sediment samples captured on the sediment floor over a 48-hour period. Each chart represents a site location; see letter at the top left right side of chart (Figure1). White areas represent seagrass beds, black areas symbolize other sources (mangrove trees + terrestrial). Green boxes around columns indicate when samples were taken in a seagrass bed. This index was not calculated for POM derived from oceanic sources because a representative surface area for the ‘ocean’ could not be defined.

**Figure 4:**
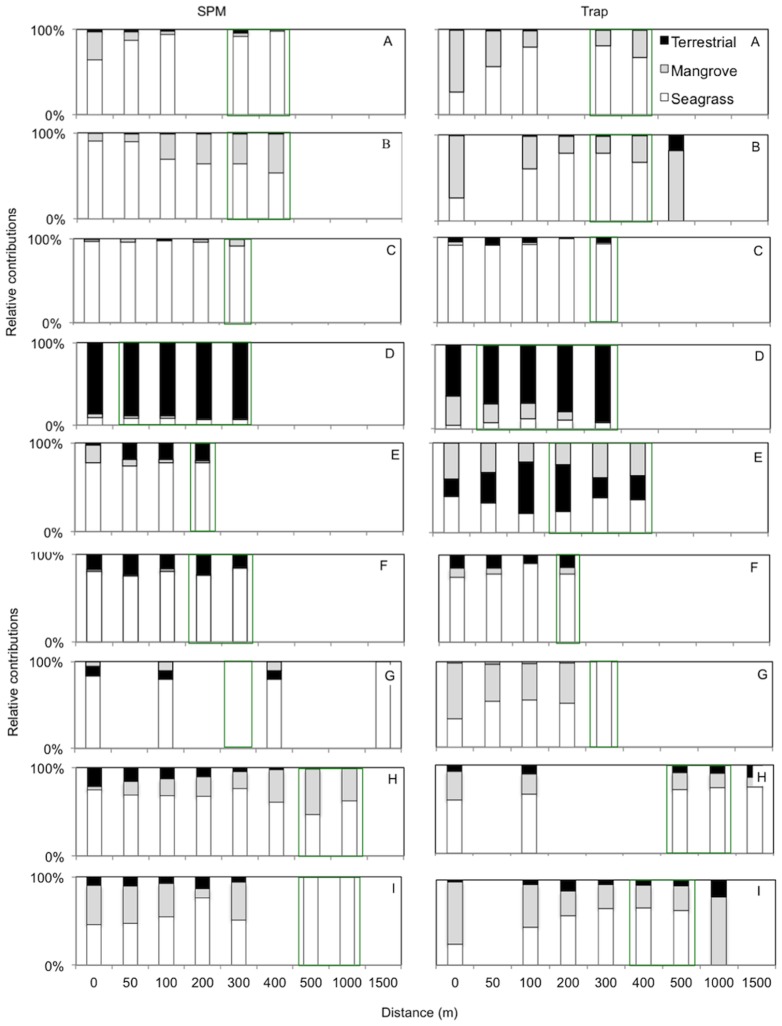
Dimensionless index indicating the relative contribution of terrestrial, mangrove and seagrass sources for suspended particulate matter (SPM) and sediment trap samples (trap), plotted in a stacked column chart. Distance (m) represents the distance along the length of the transect from the land (0 m) to the ocean for each site. SPM and trap are sample type, SPM is suspended particulate matter taken from the water column. Whilst trap represents sediment samples captured on the sediment floor over a 48 hour period. Each chart represents a site location, see letter at top left right side of chart (Figure 1). White areas represent seagrass beds, grey and black areas symbolise mangrove forests and terrestrial sources respectively. Green boxes around columns indicate when samples were taken in a seagrass bed. This index was not calculated for POM derived from oceanic sources, because no representative surface area for the ‘ocean’ could be defined.

The mixing model determined the fraction of each source end-member (mangrove and seagrass plants, terrestrial and oceanic) in the mean of 2–3 individual sediment traps and SPM samples for the three sites (Table 3). The majority of SPM samples (for sites B, C, D, F, G, H and I) showed a spatial pattern for oceanic sources, where the oceanic source contribution increased from 0 m to the end of the transect (Table 3). This pattern (for oceanic sources) was similarly repeated in six of the trap samples (sites A, B, C, D, F and I) (Table 3). The bulk of mangrove plant contributions for SPM samples showed a spatial pattern where the source influence decreased with distance from 0 m to the end of the transect (B, C, D, E, F, G, I). This same pattern was seen in the trap samples (A, B, C, D, E, H and I) (Table 3). Only approximately half of terrestrial plant contributions for SPM samples showed a gradient through the transect (C, D, H and I) (Table 3). However, over half of trap samples (A, B, C, D, G and I) did show a spatial pattern, where terrestrial plant contribution was higher nearer the landward side of the transect (Table 3). The seagrass contribution was highest in the seagrass beds for SPM samples at sites A, B, F and for trap samples at sites E and F (Figures 3, 4 and Table 3). The remainder of the trap and SPM samples showed no spatial pattern in the seagrass plant contribution (Figures 3, 4 and Table 3). For SPM samples, variation of contributions across the transect was very small (0.8–6%); and this was repeated for trap samples (0.5–12%) (Table 3).

**Table 3:** End member contributions of each source (terrestrial, mangrove, seagrass and oceanic) to each sediment sample (SPM & trap), as determined from the mixing model.

Site	Sample	Distance (m)	0	50	100	200	300	400	500	1000	1500
		Source	Contribution (%)								
A	SPM	Mangrove	17	3	1		1∧	1			
		Oceanic	66	86	90		83∧	85			
		Terrestrial	12	7	5		12∧	4			
		Seagrass	5	4	4		4∧	10			
	Trap	Mangrove	33	6	1		0.9∧	2.8∧			
		Oceanic	60	90	97		98∧	95∧			
		Terrestrial	5	3	1		0.5∧	0.8∧			
		Seagrass	2	1	1		0.6∧	0.9∧			
B	SPM	Mangrove	2	2	8	18	11∧	5∧	ns	ns	ns
		Oceanic	93	94	86	73	84∧	88∧	ns	ns	ns
		Terrestrial	1	1	3	4	3∧	4∧	ns	ns	ns
		Seagrass	3	3	3	5	2∧	3∧	ns	ns	ns
	Trap	Mangrove	65	ns	21	6	6∧	1∧	14	ns	ns
		Oceanic	22	ns	65	86	91∧	95∧	80	ns	ns
		Terrestrial	9	ns	9	4	1∧	2∧	2	ns	ns
		Seagrass	4	ns	5	4	2∧	1∧	4	ns	ns
C	SPM	Mangrove	13	10	3	11	12∧				
		Oceanic	65	78	77	67	78∧				
		Terrestrial	10	5	13	11	6∧				
		Seagrass	12	7	7	11	4∧				
	Trap	Mangrove	5	0.01	3	0.01	1∧				
		Oceanic	64	78	60	98	80∧				
		Terrestrial	27	20	33	1	17∧				
		Seagrass	4	2	4	0.9	2∧				
D	SPM	Mangrove	1	0.3∧	0.2∧	0.1∧	0.6∧				
		Oceanic	75	85∧	79∧	75∧	83∧				
		Terrestrial	23	14∧	20∧	24∧	16∧				
		Seagrass	1	0.7∧	0.8∧	0.9∧	0.4∧				
	Trap	Mangrove	11	6∧	5∧	2∧	0.2∧				
		Oceanic	52	57∧	59∧	67∧	80∧				
		Terrestrial	36	35∧	33∧	29∧	19∧				
		Seagrass	1	2∧	3∧	2∧	0.8∧				
E	SPM	Mangrove	7	1	1	0.2∧	ns	ns			
		Oceanic	79	69	34	76∧	ns	ns			
		Terrestrial	11	28	63	23∧	ns	ns			
		Seagrass	3	1	2	0.8∧	ns	ns			
	Trap	Mangrove	3	3	1	1∧	2∧	3∧			
		Oceanic	77	65	68	72∧	82∧	70∧			
		Terrestrial	17	29	29	25∧	13∧	25∧			
		Seagrass	3	3	2	2∧	3∧	2∧			
F	SPM	Mangrove	1	0.5	1	0.3∧	0.8∧				
		Oceanic	74	72	81	75∧	72∧				
		Terrestrial	24	27	18	24∧	26∧				
		Seagrass	1	1	1	1∧	2∧				
	Trap	Mangrove	7	3	0	7∧					
		Oceanic	43	65	80	30∧					
		Terrestrial	47	30	18	59∧					
		Seagrass	3	2	2	4∧					
G	SPM	Mangrove	1.0	ns	1.6	ns	ns	1.3∧			1∧
		Oceanic	74	ns	80	ns	ns	94∧			71∧
		Terrestrial	17	ns	12	ns	ns	2∧			19∧
		Seagrass	8	ns	6	ns	ns	3∧			9∧
	Trap	Mangrove	26	30	24	32	38∧				
		Oceanic	62	34	50	41	40∧				
		Terrestrial	5	17	10	9	6∧				
		Seagrass	7	19	16	18	16∧				
H	SPM	Mangrove	1	6	9	11	7	15	22∧	15∧	
		Oceanic	68	58	54	56	75	71	67∧	74∧	
		Terrestrial	23	25	24	20	7	4	3∧	1∧	
		Seagrass	8	11	13	13	11	10	8∧	10∧	
	Trap	Mangrove	20	ns	20	ns	ns	ns	16∧	8∧	7
		Oceanic	48	ns	23	ns	ns	ns	31∧	58∧	40
		Terrestrial	17	ns	34	ns	ns	ns	29∧	19∧	34
		Seagrass	15	ns	23	ns	ns	ns	24∧	15∧	19
I	SPM	Mangrove	6	4	5	1	6	ns	4∧	1∧	
		Oceanic	69	77	71	72	72	ns	76∧	82∧	
		Terrestrial	14	11	11	14	10	ns	13∧	10∧	
		Seagrass	11	8	13	13	13	ns	7∧	7∧	
	Trap	Mangrove	22	ns	9	10	3	4∧	2∧	3∧	
		Oceanic	59	ns	68	62	71	71∧	84∧	77∧	
		Terrestrial	6	ns	10	12	15	8∧	5∧	8∧	
		Seagrass	13	ns	13	16	11	17∧	9∧	12∧	

Distance (m) represents the distance along the transect from land (0 m) to the ocean for each site. SPM and trap are sample type, SPM is suspended particulate matter taken from the water column. (ns) means that the model did not find a solution. (∧) represents sediment trap and SPM samples located in seagrass beds. Blank spaces indicate that no samples were collected.

Step-wise regression indicated only two significant correlations. A negative correlation between terrestrial trap contributions (y) and catchment area (x) at 100 m (y = −0.78x+27.2, R^2^ = 0.5, p = 0.04) and a positive correlation were shown for mangrove contribution to trap samples at 0 m contributions with width of mangrove forest (y = 12.8x+2.8, R^2^ = 0.6, p = 0.03).

## Discussion

This study was designed to clarify the origin and exchange of different OM sources within tropical coastal waters. Despite the high local primary production in mangrove and seagrass stands, we found that oceanic sources dominated trap and SPM samples along the entire transect. The mixing model results however showed that mangrove forests and terrestrial sources did contribute, especially to the trap samples. Interestingly we found a correlation between the width of the mangrove forest and their contribution, which provides new insights into physical processes (such as exposure to hydrodynamics) associated with mangrove outwelling.

However when taking into account the relative size of the ecosystem (mangrove forest, catchment area and seagrass beds) we found that seagrass beds, although occupying the smallest area of all the potential sources, contributed significantly to the OM in stations along land- to seaward transects. This provides further evidence of their importance as a nutrient (dissolved and particulate) source in the tropical coastal seascape. Before moving to the implications of these findings, we first discuss the sampling and modeling approach that we undertook.

### Source contributions to organic matter

Mixing models are often used to estimate contributions to a mixture [24]–[25]. Some samples could not be solved with the model, which indicates that they violate some of the implicit assumptions of the linear mixing model formulation, such as imprecise measurement of end-members values, the existence of unidentified sources, or degradation processes that have altered the isotope or C:N ratio of the OM. For our study site, we are confident that we included the dominant POM sources in our design and the end-member values were consistent and estimated with a low uncertainty (Table 1). We therefore believe that the few samples that fell outside the mixing polygons (Figure 2) had already undergone substantial biogeochemical modification that lead to a decrease of δ^15^N values and/or N content through mineralization and denitrification [4], [28]. We think that the trap samples that fell out of the end-member polygon contained predominantly re-suspended bottom sediment (not unlikely given the close proximity to the sediment). Re-suspension of bottom sediment is a potential source of matter caught in the trap that had been subjected to biogeochemical modification.

There was a strong oceanic signal in the trap samples for the nine sites, suggesting that oceanic-derived OM settled out (Figure 2). The SPM and trap samples indicated a substantial oceanic input, which is in agreement with previously studies on coastal wetlands [29]–[30]. The OM of the trap samples at sites A, B, G and H showed a strong contribution from mangrove sources. These sites have large channels coming from the inner mangrove area, which may explain the increased contribution of mangrove OM. Sites G, H and I trap samples within the seagrass bed show an strong inclination towards the seagrass plant end-member and this, indicates that the seagrass canopy traps seagrass OM [10], [31]–[32].

### Landscape patterns of organic matter fluxes

Terrestrial-derived organic material showed an under contribution relative to the size of the catchment area. Different land uses may donate particulate and dissolved nutrients to downstream mangrove forests, but it is plausible that mangrove forests trap much of the nutrients that are flowing into them [33]–[34]. The negative correlation between catchment area and terrestrial OM is a data artefact. This has occurred due to sites A and B having catchment areas that are an order of magnitude bigger than the others, conversely sites A and B display a minor terrestrial contribution thus these two sites skew the correlation.

Mangrove derived organic matter contributed in proportion to their surface area. The latter finding may be surprising because mangrove forests are known to have a high productivity [7]–[8]. However, mangrove forests are also known to retain much of their POM and associated nutrients within the forests due to retention mechanisms as supported by other studies [33]–[35]. Our results indicate that the magnitude of outwelling of OM at the mangrove boundary (0 m) is correlated with the sea-facing width of the mangrove forest and therefore the extent that the forest is exposed to waves. Waves have been shown to decrease the trapping capacity of mangrove roots and therefore increase the transport of POM through mangrove roots [34]. The shape of the mangrove forest may have a strong influence on retention of POM. This suggests that mangrove forests that are distributed thinly along a coastline or large delta would export more POM because of their extensive frontal width, which is exposed to greater levels of hydrodynamic energy. In contrast, mangrove forests that extend inland or within protected bays may retain much of their POM and other sources of nutrients (dissolved and particulate) flowing into them. Our study sites are a mixture of these two categories category (Figure 1).

If one compares the potential amount of influx of OM sources in relation to their areal extent, seagrass is “relatively” a much more important source than mangrove forests or terrestrial sources (Figures 3 and 4). If seagrass beds are an important source of OM they may donate this material to other ecosystems and organisms. For example, stony corals have been found to assimilate particulate seagrass as a food source [36] and seagrass leaves are known to be an important food source for herbivore organisms [40]. No relationships were found between seagrass plant contribution and physical factors: hence there may be another controlling factor that has not been measured in the present study. Local hydrodynamics are a potential one. Indeed, as turbulence and wave energy can be a structuring factor in seagrass habitats. For instance, waves with an overlaying current have been found to increase litter movement through seagrass beds [34]. Herbivores digesting the seagrass plants and transferring nutrients via faeces across the tropical coastal seascape [37]–[39] may be another undetected factor.

Approximately one quarter of the samples showed a higher seagrass contribution within the bed but otherwise, the seagrass contribution seemed unrelated to the distance from or within the seagrass bed (Figures 3 and 4). This indicates that seagrasses have a spatially extended influence beyond the physical boundaries of the bed. Although the mechanisms are still unknown of what controls seagrass contribution, our results clearly show that seagrasses are a important contributor of organic matter in the tropical seascape of Phang-nga bay relative to their spatial coverage.

### Future Perspective and Management Implications

The high oceanic contribution (Table 3) shows how important oceanic derived nutrients are to ecosystems and organisms in these locations. We reason that this may be because of the high tidal flushing in all of our sites allowing for a large exchange of water. This could have implications for pollutants from industries such as fish farms being easily flushed into seagrass beds or mangrove forests from this tidal exchange.

Terrestrial input had the smallest contribution to the OM (Figure 3 and Figure 4). The differences in urbanizations across the 9 sites showed no significant correlation with terrestrial derived OM. Additionally, mangrove forests were not found to be an important input for OM in the coastal zone, compared to oceanic or seagrass organic matter. Recent research has found that mangrove forests may be an effective POM trap and therefore a strong nutrient sink [34]. Mangrove forests are effective filters, allowing them to retain most nutrients fluxes from terrestrial sources. For this reason the integrity and a critical size of the forest should always be ensured. Although many mangroves have been reduced in aerial extent in the Phuket region of Thailand, our results indicate that, though impoverished, mangroves may still maintain important ecosystem services of retaining mangrove and terrestrial derived nutrients in this region. However, continued deforestation or non-sustainable use of the forests may jeopardize this function.

An important consideration is that changes in mangrove forest aerial extent or biomass could reduce buffering and therefore increase the flux of terrestrial-derived nutrients to the coastal waters. This in turn could cause nutrient enrichment or increased turbidity for seagrass beds, causing physiological problems for the seagrass plants that could reduce the area and biomass of this ecosystem [42]. We have shown that seagrass beds are a comparatively large source of POM for adjacent ecosystems. However, seagrass beds can become unstable, for example following eutrophication [41] or hurricane damage [15]. This instability could have implications for the productivity of the seagrass beds and thus, their role as a nutrient source for other ecosystems and organisms could be affected. These alterations to the integrity of the system could have consequences on their ecosystem function as a nutrient sink or buffer for excess nutrients for sensitive adjacent ecosystems such as coral reefs. Therefore, a key role in management of these areas is to ensure the health and physical/physiological structure of seagrass beds. Both factors (health and physical/physiological structure of beds) can be related to quantities of POM being trapped and outwelled [41], [43].

This study provides further evidence that the existence of connective particulate fluxes, which occur between ecosystems in the tropical coastal seascape, do exist. These connections could have implications for strengthening management especially from an ecosystem-based perspective.
